# Book Review

**Published:** 1921-09

**Authors:** 


### Reviews of Books.

**The Early History of Surgery in Great Britain.** By G. Parker, M.A., M.D., M.R.C.S., Pp. x., 204. London: A. & C. Black Ltd. 1920. Price 7s. 6d.—Dr. Parker has added an important book to the historical literature of surgery, and at the same time established his place among the men of letters of Bristol. For this *History of Surgery* is not a mere survey of improvements in the art or technique of the operative branch of medicine, it is a study of the surgeon's share in social evolution. Dr. Parker traces out the development of the education of surgeons, their place in the social scheme, their organisation into craft-guilds or companies, and their final recognition as a profession. To the modern sociologist this evolution of a trade will prove full of interest, and the author is to be congratulated on having viewed his subject from the wider standpoint. It is by studies such as this that thoughtful statesmen are instructed when faced by aspirations of handiworkers and craftsmen of to-day to claim a higher place in the commonwealth of industry. We see in the rise of the surgeon in Great Britain a gradual upward progress from a menial and uneducated handiworker to recognition as a man of science, of polite learning and even of gentle birth. Dr. Parker shows correctly enough that there had been in earlier times surgeons of high social standing and great learning ; but when Europe was overrun by barbarians from the North science, and with it surgery, suffered total eclipse. In British and Saxon times surgery fell into ruin. He deals with the renascence of surgery in four periods or stages. First, that which followed the sudden appearance of universities and hospitals in Europe in the twelfth century ; second, that due to the great Renaissance and the elaborate educational system of the barber-surgeons in the sixteenth century ; third, that which sprang from the revival of hospitals and commencement of hospital schools and clinical teaching in the eighteenth century ; and lastly, the present period, which anæsthetics and the discoveries of Pasteur and Lister have made possible. The author says, and we agree with his judgment, that this last stage will not be discussed in his book. Our one adverse criticism of Dr. Parker's book is that he carried it a little too far into modern times. The natural ending to this volume seems to us to come at a period when the guilds and barber-surgeons companies disappear in the eighteenth century and are replaced by the hospital schools and universities. Frankly, the short biographical notices of eminent British surgeons of the eighteenth and nineteenth centuries do not seem to fit into the author's scheme, and in any subsequent edition should be omitted altogether. Dr. Parker's labours have resulted in a most valuable contribution to our knowledge of the organisation of surgery as a profession. The method is new, and so is a great deal of the material with which the story of progress is illustrated. We are told how the universities began in various towns during the Middle Ages as simple craft-guilds of students or teachers ; associations were formed of educated thinkers. Physicians were accepted as members of a learned profession, and in some universities, particularly in Italy, surgery was not divided from medicine and shared in the elevation. There were doctors of surgery as well as of medicine. Paris excluded the surgeon, and where the influence of Paris extended the practice of surgery was held to be degrading to the scholar. Oxford, deriving much of her organisation, if not her very origin, from Paris, was affected by this folly. The English universities gave little or no encouragement to the progress of the art, although they granted licences to practise. Dr. Parker traces out clearly and carefully the separation of medicine from surgery and demolishes one of our pet beliefs, that this separation was due to the decrees or influence of the Church. The definite rules of the Church as to surgery do not appear until after the fashion of the times had restricted the practice of surgery to men of low social standing. The rise of surgery into something like importance began in the twelfth and thirteenth centuries and is attributable to several causes. Among these causes may be reckoned the fashion of founding hospitals which suddenly spread throughout Europe, the increase of bathing-houses during the leprosy epidemic, with their attendant barber-surgeons, and the demand for military surgeons. Contemporaneously the social revolutions gave birth to numerous craft-guilds, which banded together the barber-surgeons of individual towns into powerful confraternities. The lesser guilds united into larger associations, so that, as in Florence, many different trades were combined into the great medical guild. The freedom of a city was a licence to practise or trade in it, and was granted only to members of a guild or company. It is in the description of the guilds and companies of surgeons and barber-surgeons that Dr. Parker is at his best. No other writer on the subject has so thoroughly studied this question, and the information as to the existence and progress of these guilds in all parts of Great Britain is new and full of interest. Military surgery played no small part in improving both the art of surgery and the organisation of the profession. The military surgeons of the sixteenth century were drawn once again from the ranks of educated men. Protests were made against the custom of sending ignorant quacks, sow-gelders and the like, as surgeons with our armies in France. Examinations were instituted and facilities for education were devised, until finally in 1540 a permanent position was given by statute to the teachers of surgery in London. Thus the Barber-Surgeons Company in London became an active educational and licensing body. This lead was soon followed in all the important towns of the kingdom. The struggles of these companies for existence and the right to train apprentices to the craft are ably described by Dr. Parker. Gradually we see the hospitals increasing in power as well as in number, until a point is reached when the teaching of surgery fell into the hands of the men who had access to the clinical material. Medical schools sprang up in connection with the larger hospitals in all parts of the country. The practice and the teaching of surgery were no longer the province of the barber-surgeon, and his place was taken by the hospital surgeon. The latter by small encroachments climbed back to his former equality with the physician, until by the end of the nineteenth century we find him once more a man of science, of polite learning, and even of gentle birth. This in brief is the story that Dr. Parker tells, and we congratulate him on the success which has attended his efforts. The illustrations lend great interest to the book, for they represent in several instances the manners and customs of the times as seen by contemporary artists. One in particular, from a fresco by Bartoli of a medieval hospital at Siena, is a work of rare beauty and art.

**The New Physiology in Surgical and General Practice.** By A. Rendle Short, F.R.C.S. Fourth Edition. Pp. xi., 291. Bristol: John Wright and Sons Ltd. 1920. Price 9s. 6d.—Nearly ten years ago, in 1911, the first edition of this book was published, and although the general arrangement remains the same, much in the present publication is devoted to problems arising out of the material presented by the wounded in the war. The chapters on food deficiency diseases, the blood and spleen, surgical shock, the spinal cord, mass reflexes and the functions of the cerebral cortex have been re-written, so that very little is left of what was to be found in the first ed tion. The book is a successful attempt to sift out from the rapidly accumulating mass of physiological work that portion which stands in close relation to the actual diagnosis of disease and treatment of patients. How far the conclusions arrived at may be taken as established and settled time only can show, but the book presents a large and carefully-thought-out contribution to our knowledge of applied physiology. The number of books dealing with this aspect of the science of physiology is but few, and this work is an accurate and interesting account of the present tendencies of physiological workers to make their observations and deductions of practical use to those whose immediate concern is the rational treatment of disease. In a future edition the work of Carlson on the hunger-contractions of the stomach might be suggested as an addition to what is given on pp. 124 and 125. The pyloric portion of the stomach apparently takes no part in these movements. The coloured frontispiece of the human prolapsed cæcum with the quiescent contracted ilio-cæcal sphincter contrasted with this orifice when relaxed gives a good idea of a part of the alimentary canal about which there was much ignorance before Mr. Short's observations. Not only the clinical worker but all interested in the relations of physiology to medicine and surgery should welcome a book such as this. It is a lucid exposition of the applications of physiology to treatment.

**Essentials of Human Physiology.** By D. Noël Paton, M.D., F.R.S. Fifth Edition. Pp. xix., 679. Edinburgh: W. Green and Son Ltd. 1920. Price 25s.—In this volume Professor Noël Paton has succeeded in bringing an excellent text-book for students up to date. The work has been written expressly for those whose ultimate aim is the practice of medicine, and particular prominence has been given to those parts of the science of physiology which bear most directly upon medical and surgical practice. The intimate association of physiology with the study of diseased conditions has been emphasised by constant reference to those disturbances of function which occur in morbid states. The book is well illustrated, and an abundant use of simple diagrams tends to make clear much that is difficult to the student. Indeed, the entire subject is handled in such a manner that the special needs of the student of medicine rather than the student of pure physiology are constantly kept in mind. A very full index of contents should prove of use when work is being revised. Professor Paton dedicates his book to his students, both past and present, from his association with whom he has derived very real pleasure during thirty-four years of active teaching.

**A Course of Practical Physiology.** By G. A. Buckmaster, M.A., D.M., and H. R. B. Hickman, M.A., M.B. Pp. vi., 138. Bristol: John Wright & Sons Ltd. 1920. Price 5s. net.—This is a very sensible little laboratory manual, covering exercises in chemical and practical physiology. It does not overdo the frog experiments, and includes some useful modern observations on the human subject, such as the various types of response to exercise—pulse-rate, breathing, and blood-pressure. There is not enough to fatigue or puzzle the student.

**A Guide to Anatomy.** By E. D. Ewart. Pp. xii., 301. London: H. K. Lewis & Co. Ltd. 1921. Price 16s. net.—This work is written for massage students and the like, and contains a clear account of all the anatomy they need know. In fact, one is tempted to observe that it is little wonder that the massage course is being gradually extended, if so much detail is required in a student's knowledge of anatomy. We are glad to find that the author, except in one instance, has resisted the temptation to use the Basle nomenclature. The illustrations are excellent; it is a great pity that many of them are marred by mistakes in spelling. Beyond a few proof-reading slips, the general get-up of the book and the index are beyond reproach. We have no doubt that those for whom it is written will find the book a great help in learning a difficult subject.

**Manual of Medicine.** By A. S. Woodwark, C.M.G., C.B.E., M.D. Second Edition. Pp. xiii., 487. London: Oxford medical Publications. 1920. Price 16s. net.—This vade-Mecum for students and practitioners, originally published in 1912, has now been revised to include the new knowledge gained during the war. The author appears to have succeeded in doing this without increasing materially the bulk of his volume, an important consideration where compactness is the chief end aimed at, as in a book of this kind.

**A Synopsis of Medicine.** By H. L. Tidy, M.A., M.D., B.Ch. (Oxon.), F.R.C.P. (Lond.). Pp. xv., 952. Bristol: John Wright & Sons Ltd. 1920. Price 25s. net.—This *Synopsis of*

*Medicine* (a companion volume to Hey Groves' *Synopsis of Surgery*) is a book which we can heartily recommend to practitioners and students. In spite of compression and brevity, it remains remarkably coherent and readable, far more so than most books of this kind. The descriptions of diseases are full and up to date. Modern views of ætiology and pathology are given considerable attention. But above all, we commend the complete and detailed consideration of treatment. The labour involved in preparing such a *Synopsis* argues a fair lapse of time since it was first taken in hand. We congratulate Dr. Tidy and the publishers on having included some very recent work, particularly on food deficiency diseases. On reading Dr. Tidy's excellent account of Trench Fever we wondered what had become of this recently wide-spread malady. Perhaps in the course of a few months a new edition will enlighten us.

**Clinical Methods.** By Robert Hutchinson, M.D., and Harry Rainer, M.D. Seventh Edition. Pp. xi., 685. London: Cassell and Company Ltd. 1920. Price 12s. 6d. net. —The new edition of this work, which has become the Bible of the clinical clerk, is revised throughout and brought up to date. Considerable additions have been made to the chapters dealing with the examination of the heart and urine and several new plates have been added. It must be difficult to keep a work of this sort, which covers so much ground, within reasonable dimensions, which it is at present; but the authors should be very much on their guard that it does not grow too large in future editions and thereby diminish its utility.

**Manson's Tropical Diseases.** By Philip Manson-Bahr, D.S.O., M.A., M.D. Seventh Edition. Pp. xvi., 960. London: Cassell and Company Limited. 1921. Price 30s. net.—Sir Patrick Manson in his preface to the first edition of this now famous volume stated that his aim was to produce a book of handy size containing adequate information. The Editor's task has been, therefore, twofold, to incorporate the numerous and important contributions of the last few years, largely a result of the experiences of the Great War, and to keep the size of the manual within the prescribed limits. The pages, it is true, are nearly the same in number, but the extra pound in weight, mainly due to an increase in size of the page, is more than compensated for by the new fount of type. Thanks to a large personal experience, Dr. Manson-Bahr has added many original observations. The volume, still indispensable to the tropical worker, will now interest the many medical men who served in the Middle or Near East. The chapter on Typhoid is entirely re-written. The account of the symptomatology and clinical course brings out very clearly the salient points of difference between typhoid and the paratyphoid infections. In view of the fact that hæmoculture is undoubtedly the most satisfactory means of diagnosis, we would have welcomed greater detail as to the duration of the bacillæmia (pp. 302 and 332), from two points of view—the earliest date on which the procedure is practicable as well as the latest, the latter often varying with the infection. The sudden onset of paratyphoid fevers has permitted of the cultivation of the bacillus in the first twenty-four hours, while in a severe enteric the bacillus may be recovered in the third week. Relapse is also a fortunate period for the bacteriologist. A curious omission is the lack of any mention of relapse in the enteric fevers. No stress is laid, and we think rightly so, on the alleged pathognomonic significance of "agglutinin fluctuation" as a means of diagnosis. The author considers the Marris atropine test the most important contribution made during the war to the diagnosis of enteric fevers. Full details of the test are given. The chapter concludes with an account of the septicæmias produced by B. Fæcalis Alkaligenes and B. Coli. The familiar plate of the unstained malarial parasite has disappeared, and five new and most instructive coloured plates of the blood picture in malaria are added. The editor favours the intramuscular route of giving quinine in severe cases, but at the same time he emphasises its undoubted disadvantages, namely the production of necrosis of tissue and the possibility of sepsis, and if given incorrectly its power to damage important nerves; these objections do not apply to the intravenous route, and the experiences in Macedonia were, on the whole, in favour of this latter method. The very vexed questions of the anti-relapse treatment of benign tertian malaria and the value of prophylactic quinine might, we consider, have been considered in greater detail. The importance of these two questions under conditions of active service in malarious countries cannot be over-estimated. The chapters on the dysenteries are store-houses of the experiences of the clinician and pathological worker during the war. In both types it is shown from the experimental and epidemiological standpoint that preventive measures should be directed mainly against the fly. The role now played by antimony and its salts in tropical disease is almost unbelievable. Such diverse affections as leishmaniasis, trypanosomiasis, schistosomiasis, dracontiasis are, if treated sufficiently early, completely cured. In each affection the drug seems to act specifically; the full details of the different schemes of dosage are given. The drug bids fair to alter the history of continents. The recent work in many other branches is treated fully and the text should be consulted by the individual worker.

We should like to call attention to the great increase in the number of illustrations, many of them unique and original. Fig. 82, p. 335, showing pneumococci in a leucocyte in the peripheral blood to illustrate the more pronounced septicæmia of pneumonias in the tropics. On page 356 a diagram indicates the means by which bilharzia ova obtain their exit from the venules ; the ingenuity on the part of the ova is nearly incredible. On pages 592–3 the life-history of Filariæ is laid bare by a series of excellent micro-photographs of the infected stegomyia pseudoscutellaris. This seventh edition cannot fail to enhance the already high reputation of its predecessors, and at the same time it demonstrates the ability of Dr. Manson-Bahr to maintain its position of authority.

**Handbook for Tuberculosis Workers.** By Noel Bardswell, M.V.O., M.D., F.R.C.P. London: John Bale, Sons and Danielsson. 1920. Price 1s. 6d. net.—This booklet of 66 pages, written by the late Superintendent of the King Edward VII. Sanatorium at Midhurst, gives the most modern views on the principal questions centring round tuberculosis. The causes and conditions which predispose to tubercle, the types of bacilli and their paths of infection, the forms of tuberculous disease and the methods by which resistance to the disease is to be established are briefly indicated, and the importance of the Ministry of Health as the central Government authority comprising many units is laid down. Of these the Dispensary and the Tuberculosis Officer is the one on whom the great responsibility lies, as he should be in touch with every case of tuberculosis in his district, and be familiar with their homes and working conditions. The many other recent developments of the day should be doing much to further the views of the National Association in preventing and curing this persisting plague.

**Medical Notes.** By Sir Thomas Horder, M.D., F.R.C.P. Pp. xi., 112. London: Oxford Medical Publications. 1921. Price 6s. net.—This little book consists of aphorisms, Sir Thomas Horder's *obiter dicta* on the diagnosis, prognosis and treatment of disease, divided into sixteen chapters. In each of these some particular area of the field of medicine is considered. It goes without saying that a series of dogmatic statements of this kind challenges a whole host of critics, but there is no physician practising who will not be grateful to Sir Thomas Horder for stating his views so fearlessly and plainly. *Creda experto ;* the book is admirably suited for reading on a railway journey.

**The Heart : Old and New Views.** By H. L. Flint, M.D. Pp. xii., 177. London : H. K. Lewis & Co. Ltd. 1921. Price 15s. net.—Dr. Flint has attempted a kind of historical survey of the development of knowledge from the earliest times till now in respect of cardio-vascular disease. His opening pages on the heart in antiquity are excellent, but when he comes to discuss the development of instrumental methods he is perhaps a little too impressed by their value. Still, he furnishes an excellent summary of this aspect of cardiology, reinforced from his own experience gleaned during the war.

**Graphic Methods in Heart Disease.** By JOHN HAY, M.D., with an introduction by SIR JAMES MACKENZIE, M.D. Second Edition. Pp. xxiii., 178. London: Oxford Medical Publications. 1921. Price 12s. 6d. net.—This work has been enlarged and brought up to date in the second edition now issued. The text is clearly printed and the subject-matter is written in a lucid and convincing manner. A great number of reproductions of polygraph tracings are given to illustrate the points in the text. The book should prove of great value to those who wish to learn how to differentiate the various irregularities of the heart-beat, and it is time that this subject became more widely known amongst practitioners, for prognosis and treatment often depends on the knowledge of the origin of these irregularities. It is our opinion that this is impossible unless the use of the Polygraph has familiarised the practitioner with the chief points of difference in the radial and jugular pulses. We cannot quite agree with the statement on page 63 that the most usual site of extra-systoles is in some portion of the primitive cardiac tube, namely the node, the A-V bundle and its many ramifications. In our experience the commoner sites are in the ventricular and auricular muscle. Chapter xi. is given up to the subject of Electro-cardiography, and is dealt with, as the author points out, very shortly. Some knowledge of this instrument and its tracings is necessary to the senior student and the practitioner, as it is not a clinical instrument, whereas the Polygraph is, and as such should be described more fully. We can highly recommend this book to practitioners who are desirous of keeping abreast with the times in their work.

**An Index of Differential Diagnosis of Main Symptoms.** By Various Writers. Edited by HERBERT FRENCH, M.A., M.D. Oxon. Second Edition. Pp. xx., 911. Bristol: John Wright & Sons. 1917.—The author claims that this volume is unique in medical literature. The book is an index in the sense that its articles on the various symptoms are arranged in alphabetical order; at the same time it is a book upon differential diagnosis in that it discusses the methods of distinguishing between the various diseases in which each individual symptom may be observed. A general index at the end gathers together the symptoms under the heading of the various diseases in which they occur. The volume aims at being of practical utility whenever difficulty arises in deciding the precise cause of any particular symptom of which a patient may complain. It is very comprehensive, covering the whole ground of medicine, surgery, gynæcology, ophthalmology, dermatology and neurology. The cordial welcome which the first edition received when published in 1912, and its subsequent reprints in 1912 and 1913, shows that this second edition, revised and enlarged, is likely to meet with increasing popularity and utility. Thirty-seven coloured plates and over three hundred illustrations in the text greatly enhance the value of the book, which is well printed and forms a handsome volume with a voluminous index of 138 pages. Twenty-two well-known contributors have assisted the editor in its production.

**Menders of the Maimed: The Anatomical and Physiological Principles underlying the treatment of Injuries to Muscles, Nerves, Bones and Joints.** By Arthur Keith, M.D., F.R.C.S., F.R.S. Pp. xii., 335. London: Oxford Medical Publications. 1919. Price 16s.—This very readable book consists very largely in biographical sketches of those anatomists, physiologists and surgeons who have contributed to the building up of our modern orthopædic surgery. John Hunter in his general recognition of the inherent powers of the living tissues towards repair of injuries, Hilton in his work on rest and pain, Hugh Owen Thomas in his mechanical contrivances for the treatment of injuries and deformities, Little, Stromeyer and Adams in relation to the introduction of tenotomy, Marshall Hall in the first enunciation of the theory of reflex nervous action in the maintenance of tonus and spasm, Duchenne in the elaboration of the physiology of movement—these great men are introduced to the reader by simple word pictures which give a graphic idea of their lives and times and the effects which their discoveries had upon contemporary teaching. The later chapters are devoted to special consideration of the mechanism and management of muscles, nerve regeneration, bone formation and the treatment of fractures, the special features of American orthopædics, massage and movements as methods of treatment, bone growth as studied by various workers from Duhamet and Ollier to Macewen, and the growth and degeneration of articular cartilage. A final chapter is devoted to bone-setting ancient and modern. There is a great wealth of information very delightfully imparted in the pages of the book, with just that amount of critical discussion of opposing theories which is necessary to guide the casual reader.

**Injuries of the Peripheral Nerves.** By Henry S. Souttar, C.B.E., M.Ch. Oxon., and Edward W. Twining. Pp. x., 152. Bristol: John Wright & Sons Ltd. 1920. Price 18s. 6d. net.

—This book, which is a valuable contribution to our knowledge of injuries of nerves, is the outcome of much work done in connection with such injuries during the war. We have learnt much from our experience of war surgery that will be of great use to us in civil practice, and in the diagnosis and treatment of injuries of nerves such lessons have been of special value. The authors have in this book called attention to many facts of great practical importance, and have placed their information clearly before us. That a work of the excellence of this one should have been written by the authors of the article on "Injuries of the Peripheral Nerves from the Surgical Standpoint" in the *British Journal of Surgery* for October, 1918, is only what might have been expected from a careful study of that article. We learn from it that up to the time of its publication the authors had observed 128 cases of injuries of nerves, and of these 70 had been operated on, and they probably added considerably to their knowledge of these injuries after its publication. When we first looked at this book we felt disappointed that the authors had not re-published in it the abstract of 122 cases which are given in their paper, but we find that 30 of them have been scattered through the text, and no doubt these are the most instructive ones. The description of the operative technique of the individual nerves is very good, and the illustrations very clear and helpful. We consider the plan which has been adopted, of indicating in the margin the subject under consideration in the text, a very helpful one. The joint authorship of the book—a surgeon and a medical officer in charge of a Physical Treatment Department—is of particular value, as it ensures a full consideration being given to such methods of treatment; and in the chapters on this subject active and passive movement, electrical treatment, massage and other methods, are dealt with in an interesting and instructive manner. The important subject of causalgia is very helpfully considered, and the record of a very interesting case is given, in which section of the antero-lateral tract in the spinal cord was employed, after all other methods of treatment (including peripheral nerve reaction) had failed, and was successful. We notice that the authors favour resection of the humerus to bridge a gap in one of the nerves of the upper arm, and they relate a successful case in which this method of treatment was adopted. They also suggest that it may with advantage be used for bridging a gap in a nerve of the forearm, "provided the nerve can be slid down sufficiently." But how can we know this until we have reached the bone? It might thus require a very extensive dissection in the course of the nerve to free it sufficiently to slide it down to the required extent. It is interesting to note what belief the authors have as to the value of nerve grafting and nerve anastomosis, as some surgeons regard them as of so little value. Of the method of grafting, in which a portion of another nerve from the same patient is used, they say the result is less certain than direct section, and slower, but they do not discard the method altogether, though they very wisely point out that every effort must be made to get direct union in preference. With regard to nerve transplantation, they admit they have had some partially successful cases, but think such cases are best dealt with by tendon transplantation. The flap method of bridging a gap in a divided nerve which the authors describe does not commend itself to us. The flap is turned down at the fibrous bulb, and although it is represented in the drawing that the nerve continuity is maintained, yet in actual practice we fear what we may call the hinge of the flap would block all nerve conduction.

**Treatment of Joint and Muscle Injuries.** By W. ROWLEY BRISTOW, M.B., B.S., F.R.C.S. Pp. xii., 148. London: Oxford Medical Publications. 1917. Price 6s.—This small book represents one of the most important aspects of the treatment of bone and joint injuries which attracted special attention during the war, but which is of constant importance in the surgery of civil life. It begins with a description of graduated muscle contraction which can be stimulated by the Faradic current, together with the special apparatus devised by the author for this purpose. It goes on to the consideration of acute and chronic sprains, of fractures, dislocations and nerve injuries. It concludes with a very short description of the rôle of massage, exercises and heat in the treatment of injuries of the limbs. The illustrations are clear and the text is simple, the argument being dogmatic rather than controversial.

**On Bone Formation: its Relation to Tension and Pressure.** By Dr. MURK JANSEN, O.B.E., Lecturer on Orthopedic Surgery, University of Leiden (Holland). Pp. 114. London: Longmans, Green & Co. 1920. Price 20s. net.—This highly specialised monograph is devoted almost entirely to a consideration of the effect of tension upon the structure and development of bones. The author holds that the commonly-accepted idea that pressure and tension have a similar effect in causing the crystallisation of the bone in lines of force is incorrect. For whilst there is abundant evidence that pressure force does produce definite lines of bone growth, there is no such evidence in relation to tension stresses. The question is discussed in relation to normal bones, *e.g.* the head and neck of the femur and the os calcis, and of abnormal bones, *e.g.* those of the ankylosed knee-joint. It is held to be proved by the evidence of sections showing the lines of bony trabeculæ that tension does not act as a stimulus to bone growth. In certain positions where the contrary appears to be the case there is reason to believe that there is an accessory pressure element, *e.g.* the pressure of a muscle or tendon, which accounts for the bony development. The excellent photographs of dried and sectioned bones form the most attractive portion of this highly abstruse work.

**Gonococcal Infection in the Male.** By NORMAN LUMB, O.B.E., M.B., B.S. Lond. Pp. xi., 328. London: John Bale, Sons, and Danielsson Ltd. 1920. Price 25s. net.—The treatment of gonorrhœa has always been looked upon as a thankless task, and it is only of recent years that any serious attempt has been made to put it upon a more satisfactory basis. The practitioner has been compelled hitherto to content himself with prescribing self-administered injections, with their inevitable result of incomplete cures. It may be said at once that the author of the book under notice has made an effectual attempt to alter this state of things. While freak treatments receive passing mention, the book mainly consists of a description—complete, clearly expressed, and admirably illustrated—of the pathology and treatment on orthodox modern lines. Special mention should be made of the excellent section on the urethroscope, with its beautifully-produced coloured plates. In the chapter on irrigation we should have liked to see included a mention of Pickin's ingenious nozzle, which is not so well known as it should be. The general get-up and the index are beyond reproach, and the book should take its place as the standard English text-book on a difficult subject.

**The Care of Human Machinery.** By R M. WILSON, M.B., Ch.B. Pp. xi., 238. London: Oxford Medical Publications 1921. Price 10s. 6d. net.—We must confess that at first we viewed with some misgiving the phrase "Human Machinery" as it is intended to be used in this book. It suggested a coldly scientific industrial system, caring for nothing but increased production of wealth and power for the Capitalist, and heedless of the health and contentment of the worker, except in so far as they minister to this production. Probably our fears are not wholly unjustified, but even if this be so, no responsibility can be laid at the door of Dr. Wilson. The whole tendency of his book indeed reassures us by showing what, fortunately, appears to be the truth—that the welfare of the worker and the interests of Capital can never really be dissociated. The book itself is a thoroughly well-written description of recent attempts to reduce costs of production by a careful study of the personality, physical and mental, of the worker. Few people realise the amount of research which has been done along these lines, the careful elaboration of the methods, the precision of the mechanical devices used, or the importance and interest from the physiological as well as the industrial point of view of the facts elicited. In our opinion, however, many medical practitioners will find it necessary in the near future to be in much closer touch with the subject than they are now. We think, too, that any keen and energetic man, anxious to get away from the beaten track, might well gain present inspiration and future profit by considering very carefully the contents of such a book as this. Some idea of the variety of the problems dealt with will be obtained if we quote a few chapter headings, *e.g.* "Hours of Industry," "Lost Time," "The Lost Worker," "Waste in Movement," "Eye-strain," "Safety First," "First Aid," "The Dust Peril," and "Noise in Industry." Tuberculosis, Venereal Disease, Ventilation and Food are also discussed. The last chapter shows what is being done in America, where a great deal of attention is being given to these matters. We conclude with one or two general impressions which occur to us after reading the book. From a clinical standpoint it is evident that more emphasis than is sometimes realised must be laid upon *nerve* fatigue, as compared with tired *muscles*. Inefficient work may result not only from the fatigue which follows long hours of work and short intervals for relaxation, but also from a certain period of re-education necessary after too much rest—a principle worth bearing in mind in other connections as well. Another conclusion seems to be that the "Works Doctor" is going to take a very important place in industry, and in our national life. His status is scarcely defined as yet, but it will probably be a combination of Medical Officer of Health, Consulting Physician, Medical Referee, and General Practitioner. At the present moment statutory provision is made to safeguard children before they are born, all through infancy, and while they are at school. Except as regards two special types of disease the chain ends quite illogically at about 14 years of age. We venture to suggest that it will be completed, not as many fear and some hope, by a State Medical Service, but by a voluntary system based upon the willing and enlightened co-operation between employers and employed for their mutual good. There are few medical men who can afford to neglect some study of a subject in which important developments are certain to take place, and it is not easy to imagine a better summary of the situation than is given by Dr. Wilson in his stimulating and suggestive book.

**Surgical Aspects of Dysentery, including Liver Abscess.** By ZACHARY COPE, M.D., M.S. Lond. London: Oxford Medical Publications. 1920. Price 12s. 6d. net.—This is a valuable contribution to surgical literature with a good index and bibliography. Summarising the contents, the author shows how amœbic typhlitis simulates appendicitis, but should not be operated upon but emetin given: moreover, operation often makes a latent dysentery acute and fatal. Dysenteric stricture, mentioned in text-books, hardly exists. Dysenteric cholecystitis should be treated by emetin. Hepatitis with leucocytosis may occur when amœbic dysentery is latent. Emetin has reduced the mortality of amœbic dysentery to 10 per cent. Arthritis is common in dysentery, chiefly of the knee and ankle. Agglutinin tests may help in diagnosis of bacillary dysentery, but vaccines and sera have little value in arthritis. There is a misprint on page 7.

**A Synopsis of Surgery.** By ERNEST W. HEY GROVES, M.S., M.D., B.Sc. Lond. Fifth edition. Pp. viii., 620. Bristol: John Wright and Sons, Ltd. Price 17s. 6d. net.—In the present edition of this popular *Synopsis* the text has been revised, some results of war surgery summarised, the figures illustrating surface markings redrawn and printed in colours, and line drawings added. For readers who require a concise and methodical account of surgery for revisionary purposes and rapid reference a better book can hardly be imagined.

**Ligations and Amputations.** By H. BROCA. Translated by ERNEST WARD, M.A., M.D., F.R.C.S. Pp. vi., 285. Bristol: John Wright and Sons, Ltd. 1917.—Professor Broca designed this book for the use of students preparing for practical examinations in operative surgery. It is confessedly based upon the teaching of Faraboeuf. The technique of the various operations is explicitly described, and the description is rendered easy to follow by the numerous illustrations provided. The latter show the position of the operator's and assistant's hands and of the instruments used in the various stages of each operation in a very clear manner. Also, in connection with each region, the anatomical facts of importance are described and illustrated. In our opinion the book will serve excellently as a guide to the student seeking to acquire skill in the performance of ligations and amputations.

**The Medical Annual, 1920.** Bristol: John Wright and Sons, Ltd. Price 15s. net.—This year's book of treatment and Practitioner's Index, issued for the thirty-eighth year, finds the world still in a state of protracted convalescence from the efforts of the great war. Questions of labour, mounting of costs, and shortness of supplies continue to cause grave anxiety. The Editor in his review of the year's work wisely points to the fact that the medical profession has not suffered from that form of malaise which seeks relief in longer hours of leisure, that their work is the result mainly of voluntary overtime efforts following strenuous daily routine, in these respects contrasting strongly with the mental condition which is now so widely epidemic throughout the world. Of the many questions mentioned by the Editor in his introduction one of the most important is the chapter (pages 416 to 427) on Public Health Administration, which includes maternity and child welfare, school medical service, industrial health and housing questions, and the colossal work of the Ministry of Public Health in general. The study of Vitamines and the whole question of Deficiency Diseases, including War Œdema and Hunger-osteopathy, have been much advanced during the year, and the dietetic treatment of Diabetes has received considerable attention. Full details of the dietetic programme advised are given by Dr. Robert Hutchinson in his article on Diabetes and he explains the rationals of the fasting method. The subject of Visceroptosis has received much attention (pages 393 to 406). The article by Mr. A. J. Walton is profusely illustrated, and deals with the whole questions of causes and treatment. The usual Dictionary of Remedies extends to thirteen pages, and the Dictionary of Treatment concludes a very useful and comprehensive volume of much new matter, well illustrated and well printed.

**Backwaters of Lethe: some Anæsthetic Notions.** By G. A. H. Barton, M.D. London: H. K. Lewis and Co., Ltd. 1920. Price 5s. net.—As the title suggests, this small book is somewhat unconventional, but it has the virtue of being couched in extremely readable language and of dealing only with matters of practical importance, the result being that prominence is given to some of the details of administration which are apt to sink into comparative insignificance in the larger text-book. Without being too dogmatic, the author gives his reasons for holding decided views on certain controversial subjects, and consequently there is special value in his references to such matters as the use of alkaloids, the dangers of light chloroform and the advantages of light ether anæsthesia, and the warming of ether vapour. The subject-matter is well selected and ably treated, and we can strongly recommend this work to all students of this special branch of study.

**The Journal of Neurology and Psychopathology.** Vol. I., No. 1, May, 1920. Bristol: John Wright and Sons, Ltd. 30s. per annum.—The main objects of this new *Journal* are to present a broad outlook on all aspects of psychological and neurological problems. It is not attached to any particular school of thought, but welcomes contributions from all, so that it may be a help in co-ordination and correlation. Interchange of opinion from different standpoints will be welcomed, where Psychological, Anatomical, Physiological and Chemical problems are all to be considered. The first part undoubtedly succeeds in fulfilling the objects aimed at. It begins with four original articles, two of which are psychological, " A Note on Suggestion" and " Thoughts about Thinking and Dreaming," the two neurological papers being on " The Treatment of Cerebrospinal Fever " and " A Case of Encephalitis Lethargica involving chiefly the Cerebral Cortex." This last article has excellent illustrations showing the pathological changes found. These articles are followed by a series of short notes and interesting clinical cases. In addition there are copious abstracts of current literature on Neuroanatomy, Neurophysiology, Neuropathology, Vegetative Neurology, Endocrinology and treatment, and also on the Psychoneuroses, Psychoses and Psychology generally in all its forms. The *Journal* promises to be one of very great value to all who wish to keep abreast of recent neurological and psychological research.

**The Industrial Clinic.** By Several Writers. Edited by EDGAR L. COLLIS, M.D. Pp. vii., 239. London : John Bale, Sons and Danielsson, Ltd. 1920. Price 10s. 6d. net.—The appearance of this volume is evidence of the remarkable extension of the scope of modern preventive medicine. Organised care of health now starts with the infant, follows the child through school life, and logically completes the scheme by State supervision of the health of the industrial worker, both male and female. The impetus given during the war by the Ministry of Munitions to the study of industrial hygiene is reflected in the nine chapters of this helpful manual, each of which is written by an acknowledged authority. The hygiene of the workshop, and all conditions of work, fatigue, food, dress, cleanliness, legal protection, and risks fall under consideration, and this small manual thus forms an indispensable guide for the factory medical officer.

**Bulletins et Memoires de la Societé Anatomique de Paris.** Tome xvi., No. 2. October and November, 1919. Paris : Maison et Cie.—The proceedings of the above-named Society as here revealed are those which would mostly in our country be the proceedings of a Pathological Society, viz. the exhibition of anatomical and pathological specimens obtained in the course of medical and surgical practice, with an account of their clinical features and treatment. Most of the specimens were obtained surgically. The communications are numerous, all interesting and instructive, and many record conditions of great rarity and of very special interest.

**The Extra Pharmacopœia of Martindale and Westcott.** Revised by W. Harrison Martindale and W. Wynn Westcott. Seventh Edition. 2 vols. London: H. K. Lewis & Co. Ltd. 1921. Price £2 4s. 6d. net.—This edition of the *Extra Pharmacopœia,* vol. ii., is about twice the size of its predecessor and rather more than twice the price. Perhaps the most striking improvement is the provision of a complete index to both volumes, identical with the one at the end of vol. i., which much enhances its value, and the book in its present form is a most useful adjunct to the laboratory library. In the section of Analytical Addenda to Materia Medica several new tests are included, notably for salicylic and acetylsalicylic acids, and the use of cadmium sulphate to increase the sensitiveness of zinc in the Marsh reaction is noted, also the organic arsenic substances are very fully dealt with; a process for the detection of hashish and a formula for a plaster mull basis are also now given. A finely-divided calomel made by precipitation is another new feature suggested as more active for local use. Caloric values of foods and a note on vitamines bring up to date the section on Nutriments. A set of tables showing the action of hydrochloric, sulphuric and nitric acids on the more common metals and their oxides supply information that is often wanted. Ionsophoresis and radiology receive a much fuller attention than before. Under processes for the detection and estimation of sugar in urine Benedict's qualitative test and Cammidge's modification are included. An innovation which will be generally welcome is a table showing the procedure to remove stains of various kinds from clothes and the skin respectively, although one of the most common stains—silver nitrate—is not included. The whole book has been rearranged as far as the order of the various sections is concerned. The sections dealing with the examination of urine, blood, fæces, water, milk, butter and stomach contents yield brief résumés which are most useful to medical man or chemist.

**Practical Obstetrics.** By E. H. Tweedy and G. T. Wrench, M.D. Fourth Edition. Pp. xxi., 557. London: Oxford Medical Publications. 1919.—We have on many occasions referred to the special value of works which are based on the practice of particular hospitals. This volume is an admirable exposition of the teaching of the Rotunda Hospital. It is in every way a sound, practical guide to the subject, not only to the student but equally so to the practitioner. The directions given for dealing with difficulties and complications arising in cases of labour in ordinary practice are particularly clear, and show how a little ingenuity and resource will enable emergencies to be met by the use of extemporised apparatus. The large experience and logical expositions of the authors will compel assent to their presentation of methods of treatment not universally accepted as correct, *e.g.* the treatment of accidental hemorrhage by the vaginal plug. In every case the method has proved a success in their hands. Space will not permit of detailed reference to all the many points to which we should like to draw attention. We will content ourselves with two in particular: (1) the views of the authors on the causation and treatment of eclampsia, and (2) Dr. Tweedy's paper on the lower uterine segment. In our opinion these should be read and carefully digested by all to whom midwifery and gynæcology have more than a passing interest. We hope that the view therein expressed may rapidly become more widely spread than at present. The book is eminently practical and well arranged, is easy to read, and is well illustrated.

**Oto-Rhino-Laryngology.** By Dr. Georges Laurens. Translated from second French edition by H. Clayton Fox, F.R.C.S.I. Pp. x., 339. Bristol: John Wright & Sons Ltd. 1919. Price 17s. 6d. net.—This book is intended as a guide to the general practitioner. The methods of examination are described in minute detail, and the practitioner is given a clear indication of "what not to do" and "what to do." While indications for operation are given, no attempt is made to describe such operations in detail unless they come within the scope of the general practitioner. The book is clearly written, and the illustrations, one of its best features, are profuse and excellent. The fact that it is a translation from the French accounts for some divergence from established British custom, and in this respect the use of the metric system in prescribing is unfortunate. The removal of enlarged tonsils by *morcellement,* here given as the method of choice, is another example. With these trifling exceptions the work can be cordially recommended.

**Anxiety Hysteria.** By C. H. L. Rixon, M.D., and D. Matthew, M.C., M.B. With a Foreword by Col. Sir A. Lisle Webb, K.B.E., C.B., C.M.G. Pp. xi., 124. London: H. K. Lewis & Co. Ltd. 1920. Price 4s. 6d. net.—The authors have set out to write a non-technical handbook for the general practitioner, and so far as war cases are concerned they have succeeded; these are, however, being more and more segregated into special hospitals, and the practitioner will not be called upon to treat so many of them in the future. A clear exposition is given of the process of complex formation and repression in the chapter on Psycho-pathology ; but the authors are somewhat hard on Freud, for after basing their whole theory on his teaching they proceed to disclaim all connection with him. The symptomatology is somewhat mixed, for obsessions are described as a symptom of anxiety hysteria after being mentioned as characteristic of psychasthenia, which is differentiated as a separate psycho-neurosis ; and again, dreams are discussed amongst conversion symptoms, which is not usual. The chapter on treatment is applicable to some war cases, but would be of very little service as a guide to the management of the civilian case.

**Anæsthetics : Their Uses and Administration.** By Dudley Wilmot Buxton, M.D., B.S. Sixth Edition. London : H. K. Lewis & Co. Ltd. 21s.—The author has taken great pains, in revising this popular text-book, to present his readers with the practical rather than with the theoretical aspects of the subject, and to avoid the introduction of unnecessary controversial matter. The new section on Shock does not profess to be a complete review of modern teaching as to the causation and pathology of the condition, but it constitutes an exceedingly clear epitome of the most generally accepted views. Among the most interesting points made in this connection are : The significance of Acidosis and the probable futility of treatment with bicarbonate of soda ; the great importance of combating any tendency to loss of body temperature ; and the need for realising that when employed too lavishly the anæsthetic drug may become a factor in the production of shock. Hemorrhage is given a new section and blood transfusion is described, but with rather too meagre a description of the technique. The chapter dealing with Ether has been recast, and includes a useful account of the rectal ether oil method—the use of chloroform and ether in fixed proportions is deprecated. We are glad to see that the importance of correct posture of the patient during anæsthesia has been duly insisted on. The book is certain to be popular, and deserves a very extensive circulation.

**Public Health Laboratory Work (Chemistry).** By Henry R. Kenwood, C.M.G., M.B., F.R.S. Edin., D.P.H., F.C.S. Seventh Edition. London : H. K. Lewis & Co. 1920. Price 15s. net.—This excellent text-book gives the necessary chemical investigations with full practical details required by M.O.H.'s and for the D.P.H. course. The selection of suitable efficient methods is scarcely more to be praised than the omission of redundant complicated tests. Only those who do the actual work can realise how much time and trouble is saved thereby. The preface states " the time has now come to exclude all but occasional references to bacteriological methods." It would have been better still to have excluded many references to parasitology, zoology and kindred sciences. Those wishing to investigate the flora and fauna of water and to identify these forms of life and the parasites of meat would have to refer to other books, therefore the pages and illustrations devoted to these subjects are out of place and useless in a book on chemistry. For instance, plates 1 to 4 and pages 64–74 have nothing to do with chemistry, and more apposite illustrations would have considerably added to the value of the book. However, this is a small matter, and no more detracts from the merits of the book than the dissertations on the beneficence of the Almighty in arranging the wonders of science did from the text-books of a century ago. Another small complaint is the mention of a proprietary preparation on page 363 for performing a test. Any preparation of a peptic ferment will answer, and it is often difficult or impossible to get special brands at short notice. There can be no doubt the book fills its requirements. The most true praise of the book was given by an examiner as a complaint. He said: "I dislike the book because it is too short, yet I find I cannot fail anyone who knows it." The omission of advertisements is an advantage in a book that is often carried about, as it reduces the bulk.

**The Theory and Practice of Nursing.** By M. A. Gullan. Pp. xv., 214. London: H. K. Lewis & Co. Ltd. 1920. Price 10s. 6d. net.—The writer of this review can claim only a slight knowledge of the literature on nursing. This little book, however, seems to fill a nitch. The book is a summary of the teaching given in St. Thomas' Hospital to probationers. In it the probationer will find a wealth of matter which will amplify and explain the mass of undigested facts that she will pick up in lectures or at the bedside. The book will be invaluable and save much time to those whose duty lies in teaching or lecturing to nurses. The arrangement of the book is delightful, and blank pages are provided for notes. Naturally there are omissions, and the book is a little old-fashioned; for instance, oxygen is administered with tube and funnel instead of by one of the newer methods, such as Haldane's apparatus. All these, however, will be corrected and brought more up to date in later editions, which should be invaluable to many generations of probationers.

**The House Fly: its life history and practical measures for its suppression.** By Major E. E. Austen, D.S.O. Pp. 52. British Museum Economic Series No. 1a. Price 1s. 6d. net.—The house fly is such a danger that the more agitation there is against it the better. This pamphlet is concise, and gives detailed instructions for destroying flies. In this it excels many others which draw horrible pictures of the dangers but do not give practical details for destroying flies. More illustrations would have been attractive, one of Col. Balfour's trap would have assisted many in producing an effective trap ; and the omission of an illustration of the fly's anatomy showing the danger of this insect's disgusting table manners owing to its vomiting at both ends like Sancho Panza, and thus spreading infections from gut and crop is disappointing, as it would have made the account of the spread of diseases which are briefly given on pages 25 to 27 more awe-inspiring. The " man in the street " is unlikely to trouble about flies unless annoyed or frightened by them. If he felt in danger of disease every time a fly settled on his food, flies would soon be scarcer than bugs in respectable houses.

**Atlas of the Sensory Cutaneous Nerves.** By WILLIAM IBBOTSON. Pp. 25. London : The Scientific Press Ltd. 1920. Price 7s. 6d. net.—This little book gives carefully-worked-out diagrams of the cutaneous nerve supply of the human body. The exact origin of every nerve trunk which supplies each area is indicated, so that the segmental as well as the peripheral supply is shown. Moreover, both the names in the old and new terminology are printed side by side. The coloured and plain diagrams are clear and well drawn, and the book supplies a simple means of reviewing many anatomical details, and is likely to be very serviceable to the busy practitioner as well as students of anatomy or of massage.

**The Prevention and Destruction of Rats.** By ELLIOT B. DEWBERRY. 1920. Price 2s. **The Rat and How to Kill Him.** By ALFRED E. MOORE. 1921. Price 9d. London : John Bale, Sons and Danielsson Ltd.—The passing of the Rats and Mice (Destruction) Act, 1919, has rightly directed popular attention to the duty as well as the desirability of controlling the economic losses and lessening the risks of disease due to the unchecked prevalence of rats. Either of these two practical little books may be depended upon for useful hints and sound advice. The former contains an introduction by Sir A. E. Shipley and the latter by the Right Hon. Lord Aberconway, P.C.

**Barrier Charts for Health Officers.** By S. H. DANEKS, O.B.E., M.B., D.P.H. London : Baillière, Tindall & Cox. 1921. Price 3s. 6d. net.—These consist of four charts printed in two colours dealing in tabular form with the principal measures employed in the prevention of communicable diseases, divided into four parts, viz. alimentary, inoculation, respiratory and contact. The four barriers round the infective foci are divided into measures dealing with sick persons and carriers, zoological factors, general hygiene and immunisation. They should prove useful in the teaching of sanitation to students, as an immense amount of information is condensed into a small space, and might be of use to Health Officers as mnemonics if suddenly presented with an unusual emergency. It would seem better to have placed the second and third barriers before the one dealing with infected persons, as that one element would only come into consideration if the two former barriers were broken down.

**Medical Electricity.** By H. Lewis Jones, M.A., M.D. Eighth edition, revised and edited by L. W. Bathurst, M.D. Pp. xv., 575. London: H. K. Lewis & Co. Ltd. 1920. Price 22s. 6d. net.—This edition of Lewis Jones' great work has been revised in the light of the experience gained during the war, and much important matter has been added. The chapter of Röntgen ray technique has been omitted, since it has become a separate and extensive subject, but a new section on ultra-violet radiation has been inserted, as well as an account of the methods of employing diathermy, and its value in surgical and medical cases. It is remarkable how many cases are now recommended for treatment by ionization or by constant currents applied to the skull in place of other methods. Thus ionization with chlorine ions is advised for the troublesome "secondary contraction" in facial paralysis, and for ulcers, otorrhœa, and septic wounds, synovitis, etc., and constant currents to the head in sleeplessness, concussion of the brain, neurasthenia and even in writer's cramp. The present editor has given much fresh information on condenser testing and the chronaxic or time duration curves of the muscles, the longitudinal reaction, the protopathic and other sensory systems, voltaic vertigo, and Bergonie's method of testing the muscles in children; but the arrangement of the book is not all that one could wish, a certain treatment is discussed in two or three places, and without cross references it is easy to miss part of the information, thus Russ' work is given on pages 291 and 295, and his name does not get into the index at all. We are glad to see that Tooth's diagrams of the segmental distribution of the nerves have been added and that Batticelli's explanation of the modes of death from electrical shock is fully set out, though a note should be given as to the long period during which artificial respiration must be continued.

**Pharmacopœia of the Queen's Hospital for Children.** Sixth edition. Pp. 76. London: H. K. Lewis and Co., Ltd. 1920. Price 4s. 6d. net.—Notable among the additions in the present issue of the Pharmacopœia is a group of emulsions in which petroleum forms the basis. Valuable rules for mothers in the management of some common ailments of children are appended. The booklet is neatly bound, clearly printed and suitable for the waistcoat pocket.

**Military Sanitation: A Handbook for Soldiers.** By Colonel Robert J. Blackham, C.B., C.M.G., M.D. Third edition. Pp. 136. London: John Bale, Sons and Danielsson, Ltd. 1920. Price 10s. 6d. net.—Four years of service in the field as an administrative medical officer have enabled the author to rewrite and to add much new matter to his book. The language is very clear and non-technical, and is intended for the unit commander of whatever rank. The subject of necessity embraces the feeding, clothing and housing of the soldier in peace and under service conditions so diverse as in the tropics and arctic regions. It is refreshing to read a book which has not descriptions of a bewildering number of types of latrines, grease traps or incinerators. What is recommended is that which has stood the test of experience in the army in France. Chapter v. deals comprehensively with the clothing of the soldier. We would have welcomed any information on the subject of impregnation of the clothing with antiseptics in view of the part played by bits of clothing in production of gas gangrene. Experimentally, very promising results have been obtained with chemicals such as tri-cresol. It is apparent that the authorities still do not consider that a soldier should be provided with any sleeping suit. The instructions as to how to inspect a kitchen or a sanitary area are very thorough and afford an inspecting officer ample opportunity for fault-finding, but we should feel inclined to congratulate any army cook who had his hands clean and his nails trimmed. We think that the part played by the fly in disease might be even more strongly emphasised. A comparison is made between the rat and the fly; and while realising that the fly is absent in the winter and that the rat is not, the only disease in temperate climates that can be definitely laid at his door is spirochætal jaundice, statistically a trifling cause of wastage in the B.E.F. Chapter ix. deals with venereal disease in the army. The commander of any unit is responsible for all measures necessary for the preservation of the health of those under him. Colonel Blackham details the methods of prevention, and gives most interesting figures as to the success of such measures. In the light of accumulated experience, it behoves any commander to make use of any method which promises to keep down the incidence of venereal diseases. The author states that an outfit is just as potent a measure of prevention of venereal disease as is quinine for malaria. His figures show that it is a far more reliable agent.

